# Simulation of the Final Size of the Evolution Curve of Coronavirus Epidemic in Morocco using the SIR Model

**DOI:** 10.1155/2020/9769267

**Published:** 2020-06-02

**Authors:** Ousama Ifguis, Mohamed El Ghozlani, Fouzia Ammou, Abdelaziz Moutcine, Zeroual Abdellah

**Affiliations:** ^1^Laboratory of Chemical Processes and Applied Materials, Faculté des Sciences et Techniques, Université Sultan Moulay Slimane, B.P. 523, Beni-Mellal, Morocco; ^2^Laboratoire de Chimie Organique et Analytiques, Faculté des Sciences et Techniques, Université Sultan Moulay Slimane, B.P. 523, Beni-Mellal, Morocco; ^3^National Higher School of Electricity and Mechanics, Casablanca, Morocco; ^4^Electrochemistry and Molecular Inorganic Materials Team, Sultan Moulay Slimane University, Faculty of Sciences and Technology, Beni-Mellal, Morocco; ^5^Molecular Modeling and Spectroscopy Research Team, Faculty of Science, Chouaïb Doukkali University, P.O. Box 20, 24000 El Jadida, Morocco

## Abstract

Since the epidemic of COVID-19 was declared in Wuhan, Hubei Province of China, and other parts of the world, several studies have been carried out over several regions to observe the development of the epidemic, to predict its duration, and to estimate its final size, using complex models such as the SEIR model or the simpler ones such as the SIR model. These studies showed that the SIR model is much more efficient than the SEIR model; therefore, we are applying this model in the Kingdom of Morocco since the appearance of the first case on 2 March 2020, with the objective of predicting the final size of the epidemic.

## 1. Introduction

During Christmas 2019, COVID-19 caused an epidemic in the city of Wuhan, Hubei Province of China [1]. It spread to the other parts of China and subsequently to many other countries around the world. Morocco is one of the countries affected by COVID-19. On 2 March 2020, the country identified the first case [2, 3], and as of 30 March 2020, confirmed cases reached up to 516 with a death toll of 27 [4]. Also, as the number of infected cases is increasing, it is necessary for modellers to estimate the severity of the epidemic in terms of the total number of people infected, the total number of confirmed cases, the total number of deaths, and basic reproduction and to predict the duration of the epidemic, the arrival of its peak, and its final size. This information can help public health agencies make informed decisions.

In this work, we used the SIR model to predict the development of the epidemic in the Kingdom of Morocco from the identification of the first case on 2 March 2020 in the city of Casablanca [2], given the reliability of the data and the definition of confirmed cases during this period and the simplicity of our forecasts and analyses. We were able to determine the detailed results of the SIR model calibration and the predictions of our model, including the distribution of the peak period, the prediction interval of future confirmed cases, and the total number of infected persons.

## 2. The SIR Model

In the SIR model, compartment *S* refers to the sensitive population in Morocco, *I* refers to the infectious population, and *R* refers to confirmed cases [5]. The latency of COVID-19 infection is biologically realistic due to an incubation period of up to 14 days; newly infected persons may not be contagious during this time period as the virus will be incubating in the organism.

Here, we highlight the difference between the latency period, the period between when an individual is infected and when he or she is infectious, and the incubation period, the period between when an individual is infected and when clinical symptoms, including fever and cough symptomatic of COVID-19, appear.

**Figure 1:**
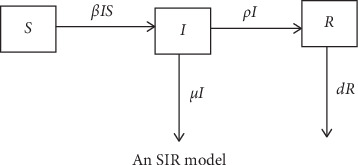
Transfer diagrams for the SIR model for COVID-19 in Morocco.

The transfer diagram for the model is shown in Figure 1. The biological significance of all model parameters is given in Table 1. A key assumption in both models is that deaths occurring in compartments *S*, *E*, and *I* are negligible during the model prediction period. The differential equation system for the SIR model is given as follows [5]:

(1) $$\begin{array}{c} \frac{\text{d}S}{\text{d}t}=-\beta SI, \end{array}$$

(2) $$\begin{array}{c} \frac{\text{d}I}{\text{d}t}=\beta SI-\gamma I, \end{array}$$

(3) $$\begin{array}{c} \frac{\text{d}I}{\text{d}t}=\gamma I. \end{array}$$

We can estimate the nature of the disease in terms of the power of infection:

(4) $$\begin{array}{c} R_{0}=\frac{\beta }{\gamma }. \end{array}$$

It is called the basic reproduction number. *R*_0_ is the average number of people infected from another person. If it is high, the probability of the pandemic is also higher.

## 3. Simulation with COVID-19 Data in Morocco

For this study, we used the data accumulated since 2 March 2020 concerning Morocco [2]. Four parameters to be estimated in the SIR model from the data are as follows: basic reproduction number (*R*_0_), contact rate (*β*), removal rate (*γ*), and final number of cases (*I*).

We do not consider the effect of the natural death or birth rate so that the total population remains constant.

- *N*=constant
- *N*_0_ = total population in Morocco = 40,000,000
- *I*_0_ = 1
- *R*_0_ = 0

## 4. Results

To solve the ordinary differential equation of the SIR model (equations (1)–(3)), we must first simulate the daily data of the SIR model, compare them with the real data, and then execute the optimization algorithm (reduce the difference between the real data and the corresponding simulated data), so that it searches for the values of *α* and *β*, which minimize the difference between the real data and the simulated ones.

In the optimal model, we supposed that the predicted infected cases should definitely be close to the actual number of infected cases; we found that the critical number of susceptible cases was about 1,812 (Table 2).

The transfer diagram for the SIR model used in the simulation of the size of COVID-19 infection in the Moroccan state is provided in detail in Figure 1.

**Figure 2:**
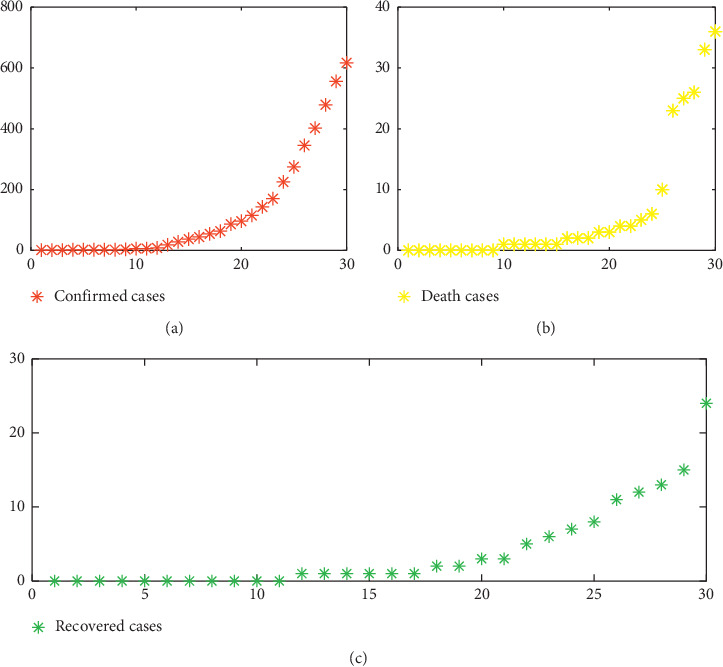
(a) Confirmed cases, (b) death cases, and (c) recovered cases from 2 March 2020 to 31 March 2020.

We note from Figure 2 that the number of confirmed cases is not very high; the highest daily confirmed number was 70 on 28 March 2020. This can be explained by the strategy of Morocco, which declared a state of health emergency and confinement on 20 March 2020, Friday, at 6 p.m., to limit the displacement of the population as much as possible, the only way to keep the coronavirus under control.

**Figure 3:**
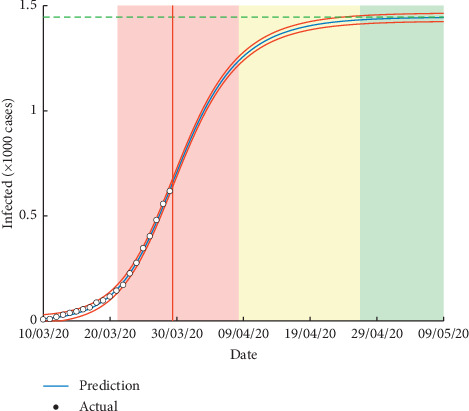
Presentation of the evolution of total infected cases (*R*_0_ = 2.003, *β* = 0.499, *γ* = 0.249, *N* = 1,812, *C*_end_ = 1,446, *S*_end_ = 366, RMSE = 7).

Figure 3, based on optimal SIR models, shows that the start of acceleration of the epidemic is around 21 March 2020, the regular growth will begin on 8 April 2020, and the end of the epidemic in Morocco would be around 26 April 2020, with a total of 1,446 infected cases and 366 final number of susceptible cases (Table 2).

**Figure 4:**
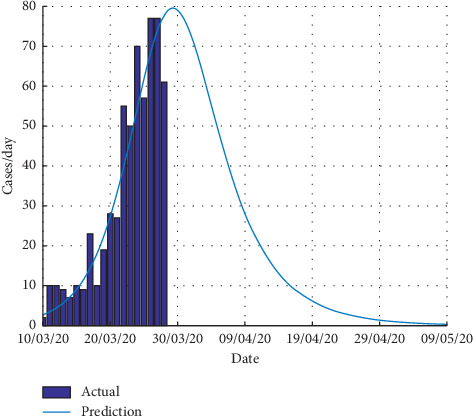
Daily estimated number of reported cases.

The peak of the reported cases will be around 28 March 2020 with 80 confirmed cases (Figure 4).

The optimal estimates of *R*_0_ ranges from 1.22 to 2.55 (Table 3); our study confirmed that the transmissibility of COVID-19 is *R*_0_ = 2.003 (Table 3), which was comparable to that of severe acute respiratory syndrome coronavirus (SARSCoV) (ranging from 2.2 to 3.7) [6] and much higher than that of Middle East respiratory syndrome coronavirus (MERS-CoV) (ranging from 0.47 to 0.91) [7].

## 5. Conclusion

Our simulation study on the optimization of the final size of COVID-19 epidemic evolution in the Kingdom of Morocco, with the SIR model, has allowed us to accurately predict the peak of the infected and death cases (Table 2), although the number of people tested is very low, about 3,079, until 31 March 2020.

The Moroccan government should probably increase the number of cases tested on a daily basis in order to accurately identify the true size of the pandemic in Morocco.

## Figures and Tables

**Table 1:** Daily increased number of COVID-19 cases in Morocco [2].

Date	Positive cases	Death cases	Reestablished cases	New cases
2 March 2020	1	0	0	1
3 March 2020	1	0	0	0
4 March 2020	1	0	0	0
5 March 2020	2	0	0	1
6 March 2020	2	0	0	0
7 March 2020	2	0	0	0
8 March 2020	2	0	0	0
9 March 2020	2	0	0	0
10 March 2020	3	1	0	1
11 March 2020	6	1	0	3
12 March 2020	6	1	1	0
13 March 2020	8	1	1	2
14 March 2020	18	1	1	10
15 March 2020	28	1	1	10
16 March 2020	37	1	1	9
17 March 2020	44	2	1	7
18 March 2020	54	2	1	10
19 March 2020	63	2	2	9
20 March 2020	86	3	2	23
21 March 2020	96	3	3	10
22 March 2020	115	4	3	19
23 March 2020	143	4	5	28
24 March 2020	170	5	6	27
25 March 2020	225	6	7	55
26 March 2020	275	10	8	50
27 March 2020	345	23	11	70
28 March 2020	402	25	12	57
29 March 2020	479	26	13	77
30 March 2020	556	33	15	77
31 March 2020	617	36	24	61

**Table 2:** The estimation of parameters of the SIR model.

Country	Initial number of cases (*I*_0_)	Contact rate (*β*)	Removal rate (*γ*)	Basic reproduction number (*R*_0_)	Critical number of susceptible cases	Final number of cases	Final number of susceptible cases
Morocco	1	0.499 (1/day)	0.249 (1/day)	2.003	1,812	1,446	366

**Table 3:** Basic reproduction number *R*_0_ (*t*) of the time-dependent SIR model of COVID-19 in Morocco based on the given data from 21 March 2020 to 31 March 2020.

Days	The reproduction number *R*_0_
21 March 2020	1,751
22 March 2020	149
23 March 2020	1,182
24 March 2020	1,229
25 March 2020	1,339
26 March 2020	1,586
27 March 2020	1,337
28 March 2020	2,559
29 March 2020	1,372

## Data Availability

The data used to support the findings of this study are cited in the article as Reference [3]. And the mathematical calculations are provided in the Supplementary Materials.

## Supplementary Materials

Supplementary Materials The mathematical calculations and also the different program steps to obtain the graphs are provided.
