# Corrigendum to “Simulation of the Final Size of the Evolution Curve of Coronavirus Epidemic in Morocco using the SIR Model”

**DOI:** 10.1155/2022/9756919

**Published:** 2022-05-31

**Authors:** Ousama Ifguis, Mohamed El Ghozlani, Fouzia Ammou, Abdelaziz Moutcine, Zeroual Abdellah

**Affiliations:** ^1^Laboratory of Chemical Processes and Applied Materials, Faculté des Sciences et Techniques, Université Sultan Moulay Slimane, B.P. 523, Beni-Mellal, Morocco; ^2^Laboratoire de Chimie Organique et Analytiques, Faculté des Sciences et Techniques, Université Sultan Moulay Slimane, B.P. 523, Beni-Mellal, Morocco; ^3^National Higher School of Electricity and Mechanics, Casablanca, Morocco; ^4^Electrochemistry and Molecular Inorganic Materials Team, Sultan Moulay Slimane University, Faculty of Sciences and Technology, Beni-Mellal, Morocco; ^5^Molecular Modeling and Spectroscopy Research Team, Faculty of Science, Chouaïb Doukkali University, P.O. Box 20, El Jadida 24000, Morocco

In the article titled “Simulation of the Final Size of the Evolution Curve of Coronavirus Epidemic in Morocco using the SIR Model” [1], the code used for this study was from the work of Prof. Batista [2, 3], which was not cited. The authors apologize for this omission.

It is also noted that the use of a simple SIR model to predict the development of COVID-19 in its early stage was limited and did not account for parameters such as containment measures.
