# Supplementary material for: Simulation of the Final Size of the Evolution Curve of Coronavirus Epidemic in Morocco using the SIR Model
Source: J Environ Public Health. 2020 Jun 2;2020:9769267. doi: 10.1155/2020/9769267 (PMC7265685; doi:10.1155/2020/9769267)
Supplement: Supplementary Materials — The mathematical calculations and also the different program steps to obtain the graphs are provided. [file 9769267.f1.docx]

The Algorithm:

function res = fitVirusCV19(getData,varargin)

%FITVIRUSCV19 SIR model for simulation of evaluation of coronavirus

%epidemy.

%

% History:

% 20/03/22 MB Created

% 20/03/23 MB Trim inital points

% 20/03/23 MB add correction for dC >= 0; add automatic selection of

% weights; correct RMSE calculation; put 3*RMSE on graph;

% add option for maxit

% 20/03/27 MB add jpg export option

%

% Usage:

% fitVirusCV19(@getData)

% fitVirusCV19(__,name,value)

%

% Input:

% getData -- A function, specified using @, that has no arguments and

% that returns vector [country,data,date0] where county is character string, data row vector

% with data, date0 initail date serial number.

%

% Optional input:

% varagin -- name/value pairs

% 'day',day -- end day (def [])

% 'plt',plt -- set plot 'on'/'off' (def 'on')

% 'jpg',jpg -- set jpg export 'on','off' (def 'off')

% 'jpres',jpres - set jpg resolution (def '-r100')

% 'maxit',maxit -- set max. number of iterations ([])

% 'nmax',nmax -- population upper limit (def 12e6)

% 'prn',prn -- set print 'on'/'off' (def 'off')

% 'w1',w1 -- weight factor for values (def [])

% 'w2',w2 -- weight factor for derivatives (def [])

%

% Optional output:

% res -- structure with results

% res.country --

% res.day -- epidemy day number

% res.date0 -- start day

% res.C -- data

% res.Ce -- forcast

% res.dif -- diference forcast-data

% res.R0 -- basic reproduction number

% res.beta -- beta

% res.gamma -- gamma

% res.N -- population size

% res.I0 -- initial number of cases;

% res.Cm -- number of case in turning point

% res.Clim -- final number of cases

% res.Cnxt -- dialy total forecase (NaN if meanless)

% res.Cinc -- daily incriment forecase (NaN if meanless)

% res.Sc -- critical nunmber of S

% res.tm -- turning point day

% res.tau1 -- duration of accelartion phase

% res.tau2 -- duration of deaceleration phase

% res.tau -- total duration

% res.tp0 -- epidemic start date

% res.tp1 -- acceleratin start date

% res.tp2 -- turning point date

% res.tp3 -- acceleratin end date

% res.tp4 -- start of ending phase date

% res.K -- final number of cases (logistic model)

% res.r -- infection rate (logistic model)

% res.A -- logistic model parameter = K/I0 - 1

% res.t2 -- initial doubling time

% res.Ca -- forcast dense curve

% res.t -- time

% res.R2 -- coefficient of determination

% res.AdjR2 -- adjusted R2;

% res.RMSE -- root maen squared error

% res.Fval -- F statistics

% res.pval --

% res.w1 -- weight for value

% res.w2 -- weight for rate

% res.fmin -- objective function value at solution (from fminsearch)

% res.flag -- exit condition: 0=OK (from fminsearch)

% res.Ca -- dense curve;

% res.t -- time for Ca;

%

%

% DISCLAIMER:

% The model may fail is some situations. In particular, the model may

% fail in the initial phase and in when additional epidemic stages or

% outbreaks (not described by SIR model) are encountered. Use it at your

% own discretion.

global C dC

global w1 w2 % optimization weights

global maxnum

%minimal check

narginchk(1,inf)

nargoutchk(0,1)

% default values

Nmax = 12e6; % max. population size

jpg = false; % set jpg export on/off

prn = false; % print results on/off

plt = true; % plot results on/off

w1 = []; % value weight

w2 = []; % derivative weight

maxnum = []; % max number of iterations

day = []; % end day

jpres = '-r100'; % plot resolution

if ~isempty(varargin)

for n = 1:2:length(varargin)

switch lower(varargin{n})

case 'jpres'

% set jpg resolution

jpres = varargin{n+1};

case 'jpg'

% set jpg export on/off

jpg = chkOnOff(varargin{n+1});

case 'day'

% set end day

day = varargin{n+1};

validateattributes(day, {'numeric'}, ...

{'>',0,'integer','scalar'});

case 'nmax'

% set upper limit for the population

Nmax = varargin{n+1};

validateattributes(Nmax, {'numeric'}, ...

{'>',0,'real','scalar'});

case 'maxit'

% set max number of iterations

maxnum = varargin{n+1};

validateattributes(maxnum, {'numeric'}, ...

{'>',999,'integer','scalar'});

case 'prn'

% set print on/off

prn = chkOnOff(varargin{n+1});

case 'plt'

% set plot on/off

plt = chkOnOff(varargin{n+1});

case 'w1'

% set weight factor for value

w1 = varargin{n+1};

validateattributes(w1, {'numeric'}, ...

{'>=',0,'real','scalar'});

case 'w2'

% set weight factor for derivative

w2 = varargin{n+1};

validateattributes(w2, {'numeric'}, ...

{'>=',0,'real','scalar'});

otherwise

error('Unknown property name %s',varargin{n})

end

end

end

% correct weights

if ~isempty(w1) && isempty(w2)

if w1 == 0

w2 = 1;

else

w2 = 0;

end

elseif isempty(w1) && ~isempty(w2)

if w2 == 0

w1 = 1;

else

w1 = 0;

end

end

% get data

[country,C,date0] = getData();

% set end day

if ~isempty(day)

nday = length(C);

if day > nday

fprintf('Invalid end day %n > %d for %s.\n', day, nday, country)

if nargout > 0

res = [];

end

% just plot data

plotData(C,date0,country,plt)

if jpg

fname = sprintf('%s%s.jpg',country,datestr(date0 + length(tt) - 1));

print(gcf,fname,'-djpeg',jpres);

end

return

end

C = C(1:day);

end

% find start

nmin = 5;

n0 = 1;

for n = 2:length(C)

if C(n-1) > C(n)

error('Invalid data C(%d)>C(%d) for %s.',n-1,n, country)

end

if C(n) == C(n-1)

n0 = n;

continue

end

break

end

if n0 == length(C)

error('Constant data set for %s.',country)

end

C = C(n0:end);

if length(C) <= nmin

error('Data set too small for %s.',country)

end

% initial guess

while true

b0 = iniGuess(C);

if isempty(b0)

if length(C) > nmin

date0 = date0 + 1;

C = C(2:end);

continue

else

break

end

end

break

end

if isempty(b0)

fprintf('Fail to obtain initial approximation for %s.\n', country)

if nargout > 0

res = [];

end

% just plot data

plotData(C,date0,country,plt)

return

end

% ... logistic curve parameters

K0 = b0(1);

r = b0(2);

A = b0(3);

C0 = K0/(A + 1);

% ... initial guess

I0 = C0;

N = 2*K0;

gamma = 2*r;

beta = 1.5*gamma;

% main calculation =======================================================%

% set infection rate and time intervals

dC = diff(C);

dC(dC<0) = 0; % correct

nday = length(C);

tt = 0:nday-1; % time span

% initial estimate

b0 = [beta gamma N I0]';

% calculate parameters

if ~isempty(w1) && ~isempty(w2)

% weigts are set by user

[b,fmin,flag] = parest(b0);

else

% automatic selection of weigths

for i = 1:3

switch i

case 1

w1 = 1;

w2 = 0;

case 2

w1 = 0;

w2 = 1;

case 3

w1 = 1;

w2 = 1;

end

[b,fmin,flag] = parest(b0);

if all(b > 0) && b(3) <= Nmax

break

end

end

end

if any(b < 0) || b(3) > Nmax

fprintf('Fail to obtain parameters for %s.\n',country)

fprintf('ini: beta = %g gamma = %g N = %g I0 = %g\n',b0)

fprintf('calc: beta = %g gamma = %g N = %g I0 = %g\n',b)

if nargout > 0

res = [];

end

plotData(C,date0,country,plt)

if jpg

fname = sprintf('%s%s.jpg',country,datestr(date0 + length(tt) - 1));

print(gcf,fname,'-djpeg',jpres);

end

return

end

% unpack results

beta = b(1);

gamma = b(2);

N = b(3);

I0 = b(4);

% postprocessing ======================================================== %

%... final value

Clim = calcClim(b);

%... value at inflection point

Cm = calcCm(b);

% basic reproduction number

R0 = beta/gamma*(1 - I0/N);

% critical number of S

Sc = gamma*N/gamma;

%... parameters of logistic model approximation

r = beta - gamma;

K = 2*(beta - gamma)/(2*beta - gamma)*N;

t2 = log(2)/r;

%... tangent slope in inflection point

k = (N - Cm)*(beta*Cm/N + gamma*log((N - Cm)/(N - I0)));

%... acceleration time

tau1 = Cm/k;

%... deceleration time

tau2 = (Clim - Cm)/k;

%... total duration of accelerated phase

tau = tau1 + tau2;

%... inflection time

tm = calcTm(b,Cm);

tm = real(tm);

%... datums

tp1 = (tm - tau1) + date0; % begin acceleration

tp2 = (tm) + date0; % turning point

tp3 = (tm + tau2) + date0; % end deceleration

tp4 = (tm + tau2) + tau + date0; % enter final phase

%... dense forcast curve

dt = 0.1;

tspan = 0:dt:2.5*tm;

warning('on')

[t,Ca] = ode45(@(t,y) odeFun(t,y,b), tspan, I0);

warning('off')

Ca = real(Ca);

%... calculate forcasting curve at data points

tspan = 0:nday; % one day more

warning('on')

[~,Ce] = ode45(@(t,y) odeFun(t,y,b), tspan, I0);

warning('off')

Ce = real(Ce);

Cnxt = Ce(end); % one day forcast

if Cnxt < C(end)

% model fails. Cnxt can not be less than current actual.

Cnxt = NaN;

end

Ce =Ce(1:end-1); % delete last

%... calculate statistics

[R2,AdjR2,RMSE,Fval,pval] = calcR2(C',Ce(1:nday));

if R2 < 0.9

fprintf('***Warning: R2 = %g\n',R2)

end

% save results =======================================================%

if nargout > 0

res.country = country;

res.day = nday;

res.date0 = date0;

res.C = C;

res.Ce = round(Ce',0);

res.dif = round(Ce' - C,0);

res.R0 = R0;

res.beta = beta;

res.gamma = gamma;

res.N = N;

res.I0 = I0;

res.Cm = Cm;

res.Clim = Clim;

res.Sc = Sc;

res.Cnxt = Cnxt;

res.Cinc = Cnxt - Ce(end);

res.tm = tm;

res.tau1 = tau1;

res.tau2 = tau2;

res.tau = tau;

res.tp0 = datestr(date0);

res.tp1 = datestr(floor(tp1));

res.tp2 = datestr(fix(tp2));

res.tp3 = datestr(ceil(tp3));

res.tp4 = datestr(ceil(tp4));

res.K = K;

res.r = r;

res.A = K/I0 - 1;

res.t2 = t2;

res.R2 = R2;

res.AdjR2 = AdjR2;

res.RMSE = RMSE;

res.Fval = Fval;

res.pval = pval;

res.w1 = w1/(w1 + w2);

res.w2 = w2/(w1 + w2);

res.fmin = fmin;

res.flag = flag;

res.Ca = Ca;

res.t = t;

end

% plot results ===========================================================%

if plt

figure

set(gcf,'Position',[100 100 832 624])

%...set scale

if max(Ca) > 1000

sf = 1000;

else

sf = 1;

end

ttt = 0:nday-2;

% plot total cases ---------------------

subplot(2,1,1)

hold on

%... plot forcast curve

plot(t + date0,Ca/sf,'LineWidth',2)

%... plot +/-SDE

nsd = 3;

h = plot(t + date0,(Ca + nsd*RMSE)/sf,'r','LineWidth',1);

h.Annotation.LegendInformation.IconDisplayStyle = 'off';

Ct = (Ca - nsd*RMSE)/sf;

Ct(Ct<0) = 0;

h = plot(t + date0,Ct,'r','LineWidth',1);

h.Annotation.LegendInformation.IconDisplayStyle = 'off';

%...get plot limits

ylm = get(gca,'Ylim'); % get y-axes limits

xlm = get(gca,'Xlim'); % get x-axes limits

www = xlm(2);

hhh = ylm(2);

%... plot cases limits

h = plot([0,t(end)] + date0,[Clim,Clim]/sf,'g--','LineWidth',1);

h.Annotation.LegendInformation.IconDisplayStyle = 'off';

%... turning line

h = plot([tm+date0,tm+date0],[0,hhh],'r','LineWidth',1);

h.Annotation.LegendInformation.IconDisplayStyle = 'off';

%... red regin

h = fill([tp1,tp3,tp3,tp1],[0 0 hhh hhh],'r',...

'FaceAlpha',0.15,'EdgeColor','none');

h.Annotation.LegendInformation.IconDisplayStyle = 'off';

%... yellow region

h = fill([tp3,tp4,tp4,tp3],[0 0 hhh hhh],'y',...

'FaceAlpha',0.15,'EdgeColor','none');

h.Annotation.LegendInformation.IconDisplayStyle = 'off';

%... green region

h = fill([tp4,www,www,tp4],[0 0 hhh hhh],'g',...

'FaceAlpha',0.15,'EdgeColor','none');

h.Annotation.LegendInformation.IconDisplayStyle = 'off';

%... add data points

scatter(tt + date0, C/sf,50,'k','filled')

h = scatter(tt + date0, C/sf,30,'w','filled');

h.Annotation.LegendInformation.IconDisplayStyle = 'off';

%... limits

xlim([t(1),t(end)]+date0);

%... what kind of thicks?

datetick('x',20,'keepticks')

%... label axes

xlabel('Date')

if sf == 1000

ylabel('Infected (x1000 cases)')

else

ylabel('Infected (cases)')

end

%... add legend

legend('Prediction','Actual','Location','best')

%... add title

tx1 = sprintf('Coronavirus epidemic in %s (SIR model): %s',...

country,datestr(date0 + length(tt) - 1));

tx2 = sprintf('%s %g %s %g %s %g %s %g %s %g %s %g %s %g',...

'R_0 = ',round(R0,3),...

'\beta = ',round(b(1),3),...

'\gamma = ',round(b(2),3),...

'N = ',round(b(3),0),...

'C_{end} = ',round(Clim,0),...

'S_{end} = ',round(N - Clim,0),...

'RMSE = ',round(RMSE,0));

title({tx1,tx2},'FontWeight','normal')

%... add grid

grid on

% plot infection rate -------------

subplot(2,1,2)

hold on

%... plot data

bar( date0 + ttt,dC)

%... plot forcasting curve

plot(t(1:end-1) + date0,diff(Ca)/dt,'LineWidth',2)

%... limits

xlim([t(1),t(end)]+date0);

%... what kind of thicks?

datetick('x',20,'keepticks')

%... add title

title('Infection rate','FontWeight','normal')

% ... add labels

ylabel('cases/day')

xlabel('Date')

%... add legend

legend('Actual','Prediction','Location','best')

%... add grid

grid on

hold off

if jpg

fname = sprintf('%s%s.jpg',country,datestr(date0 + length(tt) - 1));

print(gcf,fname,'-djpeg',jpres);

end

end

% print results ======================================================%

if prn

fprintf('Epidemic modeling by susceptible-infected-recovered (SIR) model\n');

fprintf(' Country %s\n',country);

fprintf(' Day %g\n',nday);

fprintf('Estimated the SIR model parameters\n');

fprintf(' Contact rate (beta) %g (1/day)\n',round(beta,3))

fprintf(' Removal rate (gamma) %g (1/day)\n',round(gamma,3))

fprintf(' Population size (N) %g\n',fix(N))

fprintf(' Initial number of cases (I0) %g\n',fix(I0))

fprintf('Basic reproduction number (R0) %g\n',round(R0,3));

fprintf('Critical number of susceptible %g\n',round(Sc,0));

fprintf('Final state\n');

fprintf(' Final number of cases %g\n',fix(Clim))

fprintf(' Final number of susceptibles %g\n',fix(N-Clim))

fprintf('Daily forecast for %s\n',datestr(date0 + length(tt)))

fprintf(' Total %g\n',round(Cnxt,0))

fprintf(' Increase %g\n',round(Cnxt-max(C(end),Ce(end)),0))

fprintf('Estimated logistic model parameters\n');

fprintf(' Epidemic size (K) %g (cases)\n',fix(K));

fprintf(' Epidemic rate (r) %g (1/day)\n',r)

fprintf(' Initial doubling time %g (day)\n',round(t2,1))

fprintf('Estimated duration (days)\n');

fprintf(' Turning day %g\n',round(tm,0));

fprintf(' Acceleration phase %g (days)\n',round(tau1,0))

fprintf(' Deceleration phase %g (days)\n',round(tau2,0));

fprintf(' Total duration %g (days)\n',round(tau,0));

fprintf('Estimated datums\n');

fprintf(' Outbreak %s\n',datestr(date0));

fprintf(' Start of acceleration %s\n',datestr(round(tp1,0)));

fprintf(' Turning point %s\n',datestr(round(tp2,0)));

fprintf(' Start of steady growth %s\n',datestr(round(tp3,0)));

fprintf(' Start of ending phase %s\n',datestr(round(tp4,0)));

fprintf('Statistics\n');

fprintf(' Number of observations %g\n',nday);

fprintf(' Degrees of freedom %g\n',nday - 4);

fprintf(' Root Mean Squared Error %g\n',RMSE);

fprintf(' R-Squared %g\n',round(R2,3));

fprintf(' Adjusted R-Squared %g\n',round(AdjR2,3));

fprintf(' F-statistics vs. zero model %g\n',Fval);

fprintf(' p-value %g\n',pval);

fprintf('Method\n');

fprintf(' Total cases weight %g\n',w1/(w1 + w2));

fprintf(' Infection rate weight %g\n',w2/(w1 + w2));

fprintf(' Objective function value %g\n',fmin);

fprintf(' Exit condition (1=OK) %g\n',flag);

end

end

function [b,fmin,flag] = parest(b0)

%PAREST Parameter estimation

%

% This function use MATLAB's fminsearch

%

global maxnum

warning('on')

if ~isempty(maxnum)

options = optimset('Display','off','MaxIter',maxnum,...

'MaxFunEvals',maxnum);

else

options = optimset('Display','off');

end

[b, fmin,flag] = fminsearch(@fun, b0, options);

warning('off')

end

function f = fun( par)

%FUN Optimization function

global C dC

global w1 w2

% upack parameter

I0 = par(4);

% set time span

tspan = 0:length(C)-1;

% solve ODE

try

warning('off')

[tsol,Csol] = ode45(@(t,y) odeFun(t,y,par), tspan, I0);

warning('on')

catch

f = NaN;

warning('on')

return

end

% check if calculation time equals sample time

if length(tsol) ~= length(tspan)

f = NaN;

return

end

% calculate optimization function

c1 = w1/(w1 + w2);

c2 = w2/(w1 + w2);

f1 = 0;

f2 = 0;

if c2 > 0

f2 = norm((dC' - diff(Csol)));

end

if c1 > 0

f1 = norm((C' - Csol));

end

f = c1*f1 + c2*f2;

end

function res = calcClim(par)

%CALCCLIM Calculate number of recoverd individuals after t=inf

beta = par(1);

gamma = par(2);

N = par(3);

I0 = par(4);

res = calcEndPoint(beta,gamma,I0/N)*N;

end

function res = calcCm(par)

%CALCCM Calculate number of cases at inflection point

beta = par(1);

gamma = par(2);

N = par(3);

I0 = par(4);

res = calcInflectionPoint(beta,gamma,I0/N)*N;

end

function res = calcTm(par,Cm)

%CALCTM Calculate peak time

beta = par(1);

gamma = par(2);

N = par(3);

c0 = par(4)/N;

warning('off')

res = integral(@fun,c0,Cm/N);

warning('on')

function t = fun(c)

tt = (1 - c).*(beta*c + gamma*log((1 - c)/(1 - c0)));

t = 1./tt;

end

end

function ce = calcEndPoint(beta,gamma,c0)

%CALCENDPOINT Calculate end density

ce = 1 + gamma/beta*...

flambertw(-beta*(1 - c0)*exp(-beta/gamma)/gamma);

end

function cm = calcInflectionPoint(beta,gamma,c0)

%CALCINFLECTIONPOINT Calculate inflection point for density curve

cm = 1 + (gamma/2/beta)*...

flambertw(-1, -2*beta*(1 - c0)*exp(-(1 + beta/gamma))/gamma);

end

function [R2, AdjR2, RMSE, Fval,pval] = calcR2(y,ye)

%CALCR2 Calculate the coefficient of determination

%

% Input:

% y -- actual values

% ye -- estimated values

%

% Output:

% R2 -- the coefficient of determination

% AdjR2 -- adjusted R2

%

% References:

% https://en.wikipedia.org/wiki/Coefficient_of_determination

%

n = length(y); % number of data points

p = 4; % number of explanatory terms in a model

ybar = sum(y)/n;

SStot = sum((y - ybar).^2);

SSres = sum((y - ye).^2); %

R2 = 1 - SSres/SStot;

% calculate adjusted R2

if nargout > 1

AdjR2 = 1 - (1 - R2)*(n - 1)/(n - p - 1);

end

if nargout > 2

% http://facweb.cs.depaul.edu/sjost/csc423/documents/f-test-reg.htm

SSM = sum((ye - ybar).^2); % sum of squares for regression

SSE = SSres; % sum of squares for residuals

SST = SStot; % sample variance x (n-1)

dfm = p - 1; % Corrected Degrees of Freedom for Model

dfe = n - p; %Degrees of Freedom for Error

dft = n - 1; %Corrected Degrees of Freedom Total

MSM = SSM/dfm; % Mean of Squares for Model

MSE = SSE/dfe; % Mean of Squares for Error (variance of the residuals)

%MST = SST/DFT; % Mean of Squares Total (sample variance)

RMSE = sqrt(MSE); %sqrt(SStot/(n - p)); % standard error of estimate

% calculate F statistics

Fval = MSM/MSE;

pval = fcdf(1/max(0,Fval),dfe,dfm); %????

end

end

function [b0] = iniGuess(C)

%INIGUESS Initial guess for logistic regression

% calculate initial K, r, A using data from three equidistant points

%

% Input:

% C -- data

%

% Output:

% b0 -- initial guess = [K r A]' or [] if calculation fails

b0 = [];

n = length(C);

if n <= 5

fprintf('***Warning: not enaught data.\n')

return

end

nmax = n - 5;

for i = 1:nmax

% calculate time interval for equidistant points: k-2*m, k-m, k

if mod(n-i+1,2) == 0

k1 = i;

k3 = n-1;

else

k1 = i;

k3 = n;

end

k2 = (k1 + k3)/2;

m = k2 - k1 -1;

if k1 <1 || k2 < 1 || k3 < 1 || m < 1

break

end

if isnan(C(k1)) || isnan(C(k2)) || isnan(C(k3))

continue

end

% calculate K, r, A ...

%.. calculate K

q = C(k2)^2 - C(k3)*C(k1);

if q <= 0

% fprintf('***Warning: iniGuess q = %g k1 = %d k2= %d k3 = %d \n',...

% q, k1, k2, k3)

continue

end

p = C(k1)*C(k2) - 2*C(k1)*C(k3) + C(k2)*C(k3);

if p <= 0

% fprintf('***Warning: iniGuess p = %g\n',p)

continue

end

K = C(k2)*p/q;

% ... calculate r

r = log(C(k3)*(C(k2) - C(k1))/C(k1)/(C(k3) - C(k2)))/m;

if r < 0

% fprintf('***Warning: iniGuess r = %g\n',r)

continue

end

%... calculate A

A = (C(k3) - C(k2))*(C(k2) - C(k1))/q*...

(C(k3)*(C(k2) - C(k1))/C(k1)/(C(k3) - C(k2)))^((k3-m)/m);

if A <= 0

% fprintf('***Warning: iniGuess A = %g\n',r)

continue

end

% this is initial guess

b0 = [K r A]';

break

end

end

function dCdt = odeFun(~,C,par)

%ODEFUN SIR model

% unpack parameters

beta = par(1);

gamma = par(2);

N = par(3);

I0 = par(4);

% set temp. vars

c0 = I0/N;

c = C/N;

% setup equation

dCdt = N*(1 - c)*(beta*c + gamma*log((1 - c)/(1 - c0)));

end

function ret = chkOnOff(inp)

%CHKONOF Check for on/off input

%

%Input:

% inp -- 'on'/'off', or 1/0 or true/false

%

%Output;

% ret -- true if inp is 'on', 1 or true, otherwise false

%

try

validateattributes(inp, {'char'},{'nonempty'});

switch lower(inp)

case 'on'

ret = true;

case 'off'

ret = false;

otherwise

error('Invalid option value')

end

catch

try

ret = inp;

validateattributes(ret, {'numeric'},...

{'>=',0,'<=',1,'integer','scalar'});

if ret == 1

ret = true;

else

ret = false;

end

catch

ret = inp;

validateattributes(ret, {'logical'},{'scalar'});

end

end

end

function plotData(C,date0,country,plt)

if ~plt

return

end

t = 0:length(C) - 1;

figure

set(gcf,'Position',[100 100 800 600])

%...set scale

if max(C) > 1000

sf = 1000;

else

sf = 1;

end

% plot total cases ---------------------

subplot(2,1,1)

hold on

%... plot curve

plot(t + date0,C/sf,'LineWidth',2)

%... add data points

scatter(t + date0, C/sf,50,'k','filled')

%... limits

xlim([t(1),t(end)]+date0);

%... what kind of thicks?

datetick('x',20,'keepticks')

%... label axes

xlabel('Date')

if sf == 1000

ylabel('Infected (x1000 cases)')

else

ylabel('Infected (cases)')

end

%... add legend

%... add title

% tx1 = sprintf('Coronavirus epidemic in %s',country);

tx1 = sprintf('Coronavirus epidemic in %s (SIR model): %s',...

country,datestr(date0 + length(t) - 1));

title(tx1,'FontWeight','normal')

%... add grid

grid on

% plot infection rate -------------

subplot(2,1,2)

hold on

%... plot data

bar(date0 + t(1:end-1),diff(C))

%... limits

xlim([t(1),t(end)]+date0);

%... what kind of thicks?

datetick('x',20)

%... add title

title('Infection rate','FontWeight','normal')

% ... add labels

ylabel('cases/day')

xlabel('Date')

%... add grid

grid on

hold off

end

function z = flambertw(n,x)

if license('test','symbolic_toolbox')

if nargin == 1

z = lambertw(n);

else

z = lambertw(n,x);

end

return

end

disp('ups')

if nargin == 1

x = n;

n = 0;

end

zmax = 1e6;

z0 = -1/exp(1)+10*eps;

switch n

case -1

if x < z0 || x > 0

z = NaN;

elseif x == 0

z = inf;

elseif x < 0

z = fzero(@fun,[-zmax,-1]);

end

case 0

if x < z0

z = NaN;

elseif x == 0

z = 0;

elseif x < 0

z = fzero(@fun,[-1,0]);

else

z = fzero(@fun,[0,zmax]);

end

otherwise

error('**flamberw: invalid n = %g.',n)

end

function z = fun(y)

z = y*exp(y) - x;

end

end
